# Mobile Thrombus Observed Around an Impella Device

**DOI:** 10.7759/cureus.70399

**Published:** 2024-09-28

**Authors:** Toru Miyoshi, Takashi Nishimura, Yu Hiasa, Hironori Izutani, Osamu Yamaguchi

**Affiliations:** 1 Department of Cardiology, Pulmonology, Hypertension, and Nephrology, Ehime University Graduate School of Medicine, Toon, JPN; 2 Department of Cardiovascular Surgery, Ehime University Graduate School of Medicine, Toon, JPN

**Keywords:** ascending aorta thrombosis, cardiogenic shock, impella, left ventricular assist device (lvad), transesophageal echocardiography

## Abstract

An Impella device (Abiomed, Inc., Danvers, Massachusetts, United States) is a useful percutaneous left ventricular assist device for patients with cardiogenic shock. However, there are cases in which patients are unable to escape cardiogenic shock and become Impella-dependent. Long-term management can lead to thrombus. A man in his thirties experienced acute myocardial infarction necessitating Impella 5.5 support. Initially, a thrombus was not detected in the ascending aorta. Nonetheless, a mobile thrombus was observed post-Impella removal. His computed tomography revealed thrombosis in the iliac artery the following day. Diligent monitoring during Impella removal is imperative.

## Introduction

An Impella device (Abiomed, Inc., Danvers, Massachusetts, United States) is a percutaneous left ventricular assist device (LVAD) featuring a small axial flow pump integrated within a catheter. This device facilitates left ventricular unloading by taking blood from the catheter tip placed inside the ventricle and expelling it into the ascending aorta [1]. It is indicated for severe heart failure with cardiogenic shock [2]. It can be removed to wean the patient off cardiogenic shock in some cases; some patients with advanced heart failure develop a dependence on it. In such cases, the duration of Impella implantation is longer, and sometimes the patient may be moved to an implantable ventricular assist device (VAD) implantation. Here, we present a case of a patient with severe heart failure who encountered difficulties in weaning themselves off an Impella device. Following the removal of the Impella device, the patient developed a mobile thrombus in the ascending aorta.

## Case presentation

**Figure 1:**
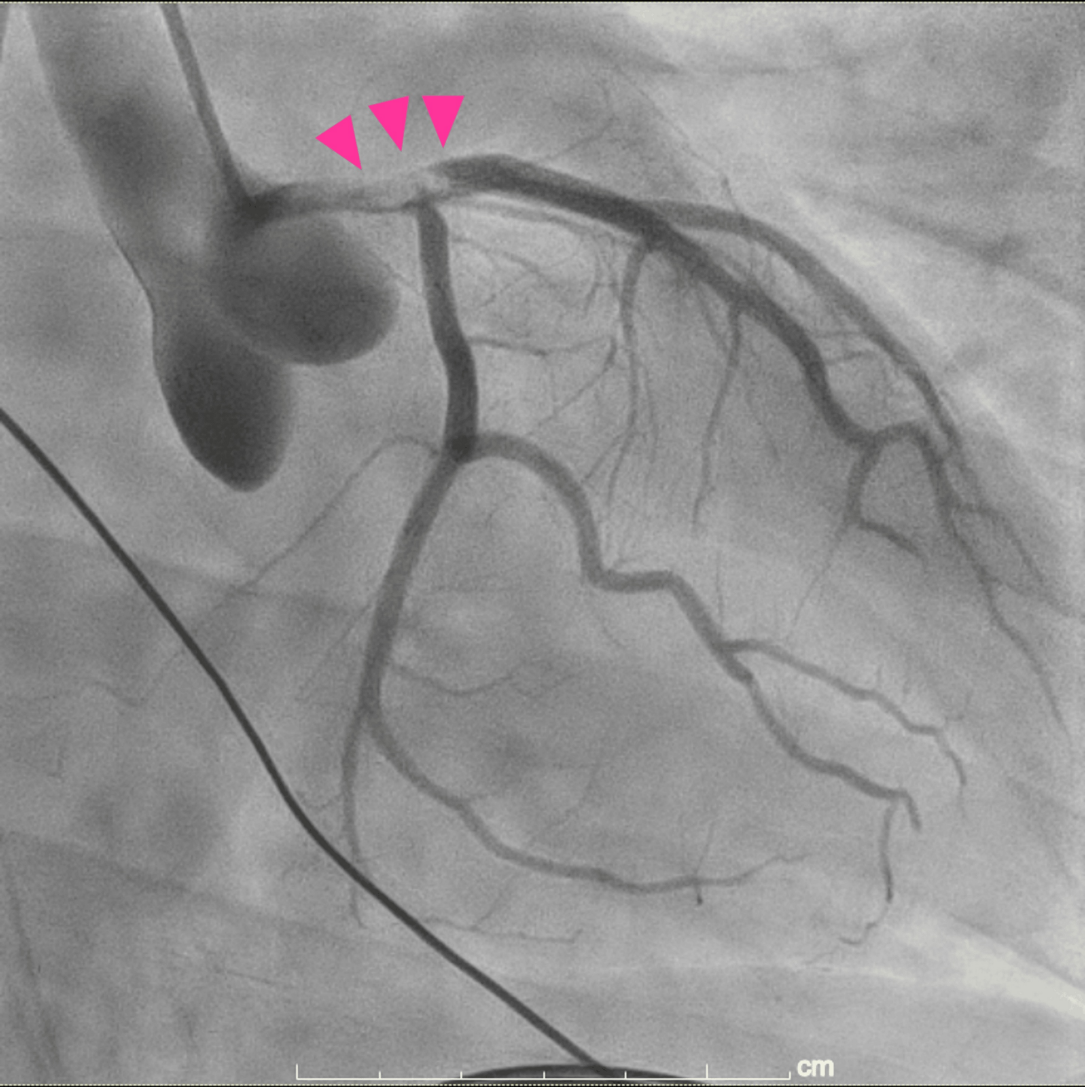
Coronary angiography Initial coronary angiography revealed a massive thrombus extending from the left main trunk to the proximal segment of the left anterior descending artery.

A man in his thirties experienced an acute myocardial infarction, with the left main trunk identified as the culprit lesion (Figure 1).

**Figure 2:**
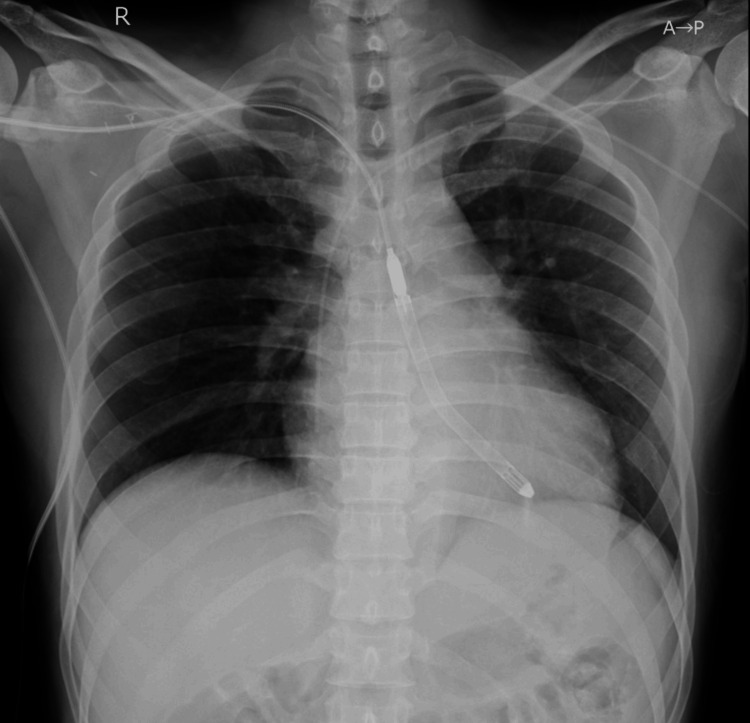
Chest X-ray His chest X-ray showed that the Impella 5.5* was inserted through the right axillary approach. No congestion was observed. *Abiomed, Inc., Danvers, Massachusetts, United States

He underwent percutaneous coronary intervention, achieving a door-to-balloon time of 50 minutes and thrombolysis in myocardial infarction (TIMI) 3 flow. However, his peak creatine kinase (CK) was 13,553 U/L and his peak CK-MB was 796 U/L. He subsequently required support from the Impella 5.5 device (Figure 2).

Discontinuing the Impella device was difficult, and he was registered on the heart transplant registry. We decided to implant an implantable LVAD (HeartMate 3^TM^, Abbott Laboratories, Chicago, Illinois, United States) as a bridge to heart transplantation. During Impella support, an anticoagulation therapy with heparin was administered, maintaining an activated clotting time (ACT) of 160-180 seconds, and activated partial thromboplastin time (aPTT) of 50-70 seconds. The most recent Impella 5.5 support lasted for 51 days, with auxiliary flow managed at P6-P8 (4.0 - 5.0 L/minute). The Impella purge flow was maintained at 11-14 ml/hour, with a purge pressure of 400-500 mmHg. No significant thrombocytopenia or elevated lactate dehydrogenase (LD) levels were observed while awaiting the implantable VAD, and tests for heparin-induced thrombocytopenia (HIT) antibodies were negative.

**Figure 3:**
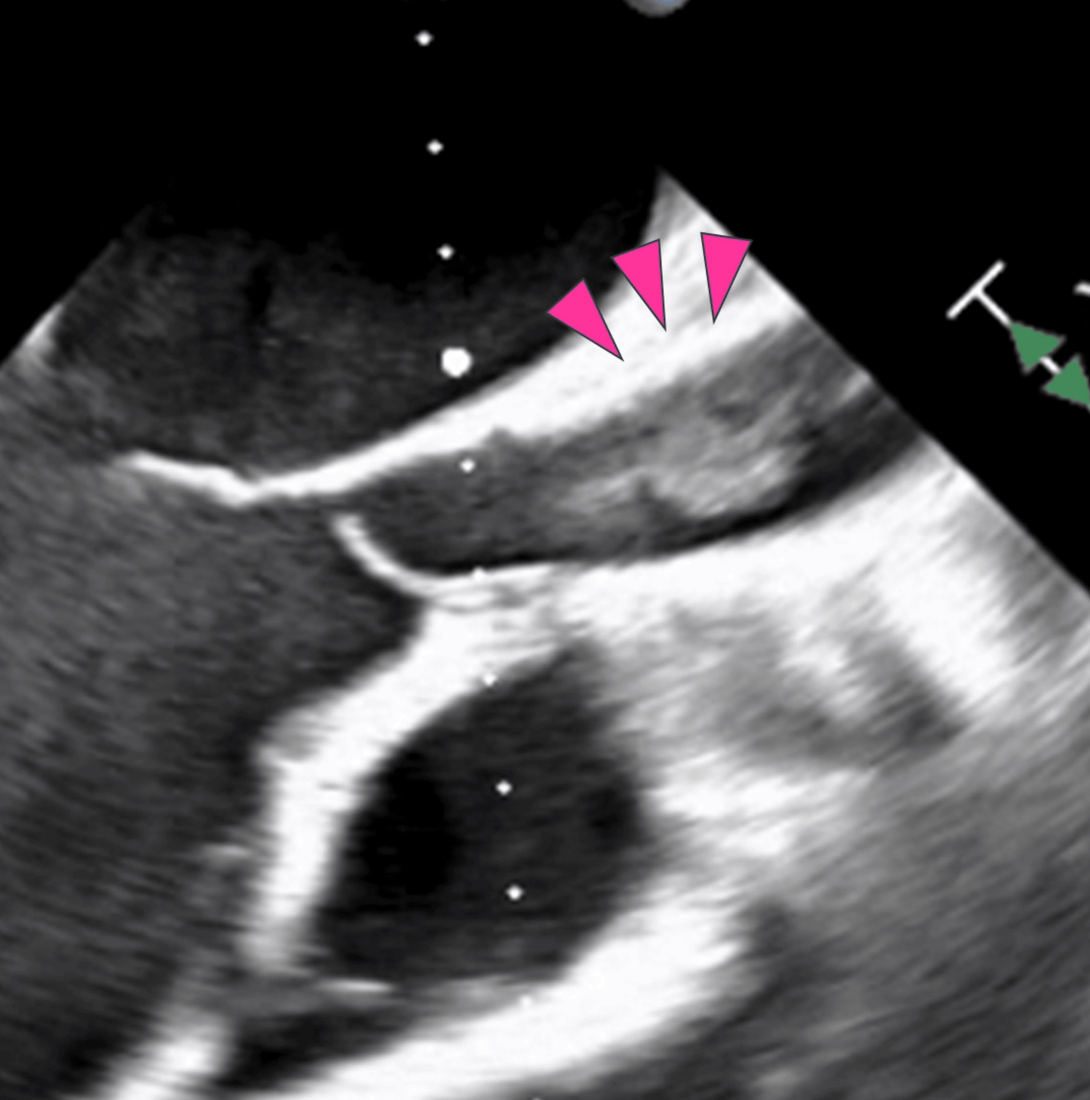
Transesophageal echocardiography A mobile thrombus was observed in the ascending aorta after the removal of Impella 5.5*. *Abiomed, Inc., Danvers, Massachusetts, United States

The initial transesophageal echocardiography (TEE) during Impella 5.5 implantation showed no thrombus in the ascending aorta. However, after Impella 5.5 removal, a mobile thrombus was observed in the ascending aorta (Figure 3).

**Figure 4:**
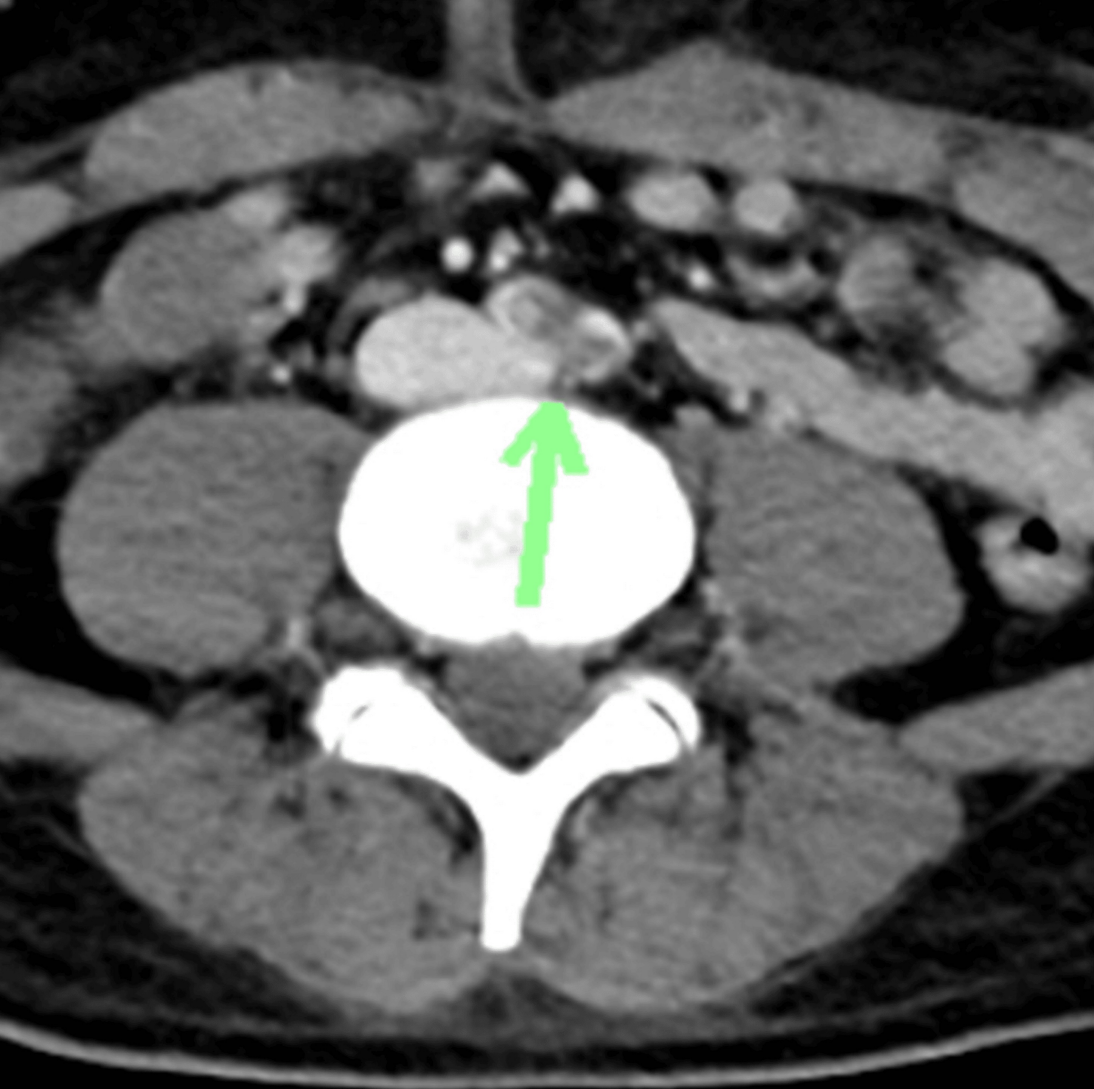
Contrast-enhanced computed tomography Contrast-enhanced computed tomography revealed a contrast defect at the bilateral common iliac arteries, suggesting a thrombus (arrows).

Immediate clamping of the ascending aorta and suction were performed. As a bridge to transplantation, a HeartMate 3^TM^ device was implanted. The following day, contrast-enhanced computed tomography revealed a contrast defect in the bilateral common iliac arteries, suggesting the presence of a thrombus (Figure 4).

Thrombi in the bilateral common iliac arteries resolved after conservative management and continued anticoagulation. There were no postoperative neurological findings indicative of cerebral infarction.

## Discussion

This report describes a case of thrombus formation in the ascending aorta during long-term Impella management. Thrombi have previously been reported in the left ventricle and descending aorta during Impella use [3-4]. With the routine use of the Impella 5.5, which offers increased durability compared to the Impella 5.0, long-term management of cardiogenic shock has become less invasive due to the reduced need for frequent device changes. However, the tendency for thrombus formation may still emerge during prolonged use.

Past reports on ventricular assist devices indicate that the ascending aorta is prone to thrombus formation due to retrograde blood flow to this area [5-6]. During Impella support, blood flow stasis over the aortic valve may be prevented by ensuring that the valve opens. However, this is often difficult in patients who become dependent on Impella support. Therefore, it is advisable to maintain vigilance for thrombus formation, using techniques such as transesophageal echocardiography, when removing the Impella device in patients who have required long-term management.

Intraoperative TEE or transthoracic echocardiography (TTE) are valuable tools for detecting left ventricular and inflow thrombi in patients supported by an Impella device. However, as demonstrated in the present case, early detection of such thrombi can be challenging. Additionally, in cases where a patient transitions to an implantable LVAD following the removal of an Impella device after prolonged support, the possibility of thrombus formation should be considered. Along with left ventricular thrombus, clinicians should be prepared for the early clamping of the ascending aorta during Impella removal [7].

## Conclusions

We experienced a case of a floating thrombus in the ascending aorta during the extubation of an Impella device. This might represent an event that had previously gone unrecognized. During prolonged management with an Impella device, it is essential to exercise vigilance within the left ventricle and the ascending aorta during device removal. The utilization of TEE during the removal of an Impella device may be advisable.
